# The information sources and journals consulted or read by UK paediatricians to inform their clinical practice and those which they consider important: a questionnaire survey

**DOI:** 10.1186/1471-2431-7-1

**Published:** 2007-01-15

**Authors:** Teresa H Jones, Steve Hanney, Martin J Buxton

**Affiliations:** 1 Health Economics Research Group, Brunel University, Uxbridge UB8 3PH, UK

## Abstract

**Background:**

Implementation of health research findings is important for medicine to be evidence-based. Previous studies have found variation in the information sources thought to be of greatest importance to clinicians but publication in peer-reviewed journals is the traditional route for dissemination of research findings. There is debate about whether the impact made on clinicians should be considered as part of the evaluation of research outputs. We aimed to determine first which information sources are generally most consulted by paediatricians to inform their clinical practice, and which sources they considered most important, and second, how many and which peer-reviewed journals they read.

**Methods:**

We enquired, by questionnaire survey, about the information sources and academic journals that UK medical paediatric specialists generally consulted, attended or read and considered important to their clinical practice.

**Results:**

The same three information sources – professional meetings & conferences, peer-reviewed journals and medical colleagues – were, overall, the most consulted or attended and ranked the most important. No one information source was found to be of greatest importance to all groups of paediatricians. Journals were widely read by all groups, but the proportion ranking them first in importance as an information source ranged from 10% to 46%. The number of journals read varied between the groups, but *Archives of Disease in Childhood *and *BMJ *were the most read journals in all groups. Six out of the seven journals previously identified as containing best paediatric evidence are the most widely read overall by UK paediatricians, however, only the two most prominent are widely read by those based in the community.

**Conclusion:**

No one information source is dominant, therefore a variety of approaches to Continuing Professional Development and the dissemination of research findings to paediatricians should be used. Journals are an important information source. A small number of key ones can be identified and such analysis could provide valuable additional input into the evaluation of clinical research outputs.

## Background

If medicine is to be evidence-based then health research findings need to be implemented appropriately in the clinical setting. However, there is an ever-expanding wealth of biomedical knowledge to be assimilated and used by clinicians [1,2]. The range of potentially available information sources is large and even for one of them, peer-reviewed journals, the choice within a specialty is enormous.

A review of the information sources used and favoured by clinicians from many different medical specialties, as well as some nursing groups, found results varied from one study to another [3]. Overall, however, other medical colleagues were the preferred source, for example Cullen found that family practitioners referred most frequently to medical specialists [4]. In contrast, others found printed materials to be the favourite source [5-7]. Even studies on the implementation of a specific clinical advance show that a wide range of sources can all play a role [8].

Despite some doubts [9], journals continue to be considered an important information source by many clinicians. Journals are also still the principal medium used to publish research findings. Assessment of both research and journals can be contentious [10-15] and in the UK, there are moves within research assessment towards giving some recognition to the impact made by research [16,17]. A key issue for researchers wishing to make their findings known to clinicians might be "Which information sources and journals are clinicians most likely to access and take notice of?"

These issues may well differ between specialties. Even within one specialty there are likely to be differences between different groups. The availability of different information sources, as reported by trainees-on-call, has been studied in relation to paediatric and neonatal units in UK hospitals [7]. The survey reported here covers the general use of information sources by paediatricians, at consultant and non-consultant career grades (NCCG) within both hospital and community environments. We aimed to determine which sources are consulted or attended by UK paediatricians to inform their clinical practice and which are considered important. We also aimed to identify how many and which specific journals were read by clinicians. This allows comparisons with the journals containing the best paediatric evidence identified by Birken et al [18] and potentially provides information that could contribute to the assessment of research outputs.

## Methods

Ethics approval was not required for this study as the survey was conducted anonymously using a list of names and addresses taken from the Medical Directory (see below) which is available in the public domain. Prior to the release of the Medical Directory, those listed are given the opportunity to exclude their names from external surveys.

The method used involved a questionnaire survey followed by analysis, comparisons within the specialty and further comparisons with other similar or related studies.

### Questionnaire recipients

Paediatricians' names and addresses were taken from the Medical Directory 2003/4 CD-ROM (produced by Informa Healthcare, UK in association with the Royal Society of Medicine, London) if they had full registration and were not retired. All doctors with a UK address specialising in paediatrics were included in the questionnaire survey unless they had excluded their names under the Medical Directory's privacy policy.

### Questionnaire structure [see Additional file 1]

The questionnaire focused first on information sources in general and then concentrated on journals.

A list of 11 information sources was presented in the questionnaire. In compiling this list, advice was sought from members of the Royal College of Paediatrics and Child Health to ensure the inclusion of information sources likely to be available to community-based or hospital-based paediatricians. Questionnaire recipients were asked to tick any information sources that they consulted or attended to inform their clinical practice. They were invited to add and tick any that were not listed and from the complete list to rank the top three.

A list of journals was constructed including general medical, paediatric and sub-specialty journals either if they contained a large number of NHS funded paediatrics papers or if they scored highly on the impact factors developed by the Institute of Scientific Information (ISI, now part of Thomson Scientific). Thus the list was derived from two sources. Firstly from the Research Outputs Database (ROD) [19] and secondly from the Journal Citation Reports (JCR) 2002 from ISI [20] [see Additional file 1]. After overlaps were removed, the two sources resulted in a total of 39 journals that were listed in the questionnaire.

The questionnaire recipients were asked to tick up to ten journals in total that they read or consulted on a regular basis to inform their clinical practice. They were invited to add any that were not listed.

Further questions related to the position they held, the number of academic and clinical sessions they worked and their predominant role.

### Questionnaire analysis

The data from the questionnaire survey were entered into a database for analysis using a double-entry procedure to ensure the integrity of the data. The names of journals added to the questionnaires by respondents were verified using Ulrich's International Periodicals Directory [21] or the internet.

The paediatricians' responses were collated and tabulated according to three criteria:

▪ their position (i.e. consultant or non-consultant career grade)

▪ whether or not they had academic responsibilities

▪ their predominant role (i.e. community-based, District General Hospital-based (DGH), working at the tertiary level)

A statistical analysis was undertaken to investigate the null hypotheses that the information sources accessed and the number of journals read were independent of the paediatrician's characteristics.

## Results

2,330 questionnaires were distributed and 993(43%) paediatricians responded. Paediatricians who had ticked more than one predominant role out of the three options totalled less than 3% in all cases and were included in both groups for analysis. The characteristics of the respondents can be found in Table 1.

### Information sources consulted or attended by respondent paediatricians to inform their clinical practice and those considered important

**Figure 1:**
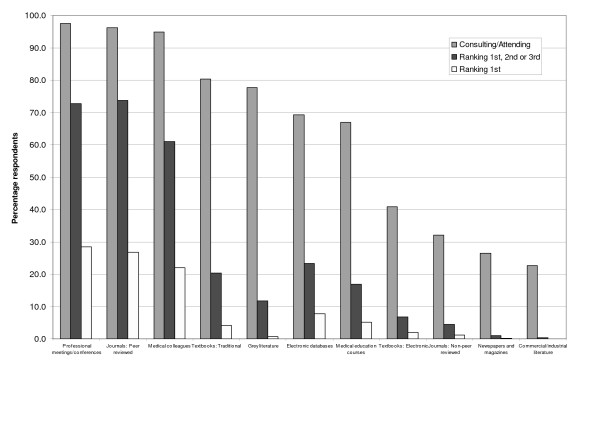
**The importance of selected information sources**. The percentage of respondent paediatricians consulting or attending selected information sources and ranking them for importance in informing their clinical practice.

Figure 1 shows the results for paediatricians overall by giving three numbers for each information source: the percentage consulting or attending it; the percentage ranking it either first, second or third; and the percentage ranking it first. Overall, the information sources perceived to be of greatest importance to paediatricians' clinical practice are professional meetings & conferences, peer-reviewed journals and medical colleagues. This picture is repeated in all three of the measures used.

**Figure 2:**
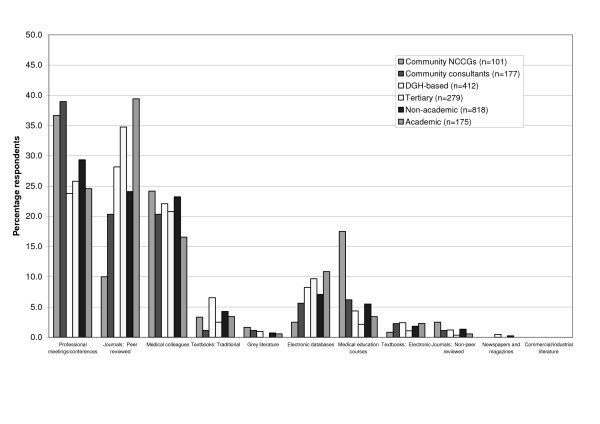
**Respondent paediatricians (%) ranking selected information sources first in importance to inform their clinical practice**. Position, academic responsibility and predominant role.

The percentage of paediatricians in different groups ranking information sources first in importance to their clinical practice is presented in Figure 2. The data are arranged to allow comparisons between: those holding different positions (the numbers with NCCG status based in hospitals were too small for analysis); those with and those without academic responsibilities; and those with different predominant roles. When comparing the distributions across the most prominent three information sources, significant differences were found at the 95% confidence level for: academics versus non-academics (Chi-square test:χ22
 MathType@MTEF@5@5@+=feaafiart1ev1aaatCvAUfKttLearuWrP9MDH5MBPbIqV92AaeXatLxBI9gBaebbnrfifHhDYfgasaacH8akY=wiFfYdH8Gipec8Eeeu0xXdbba9frFj0=OqFfea0dXdd9vqai=hGuQ8kuc9pgc9s8qqaq=dirpe0xb9q8qiLsFr0=vr0=vr0dc8meaabaqaciaacaGaaeqabaqabeGadaaakeaacqaHhpWydaqhaaWcbaGaeGOmaidabaGaeGOmaidaaaaa@3074@ = 16.12, p = < 0.001); and for community-based consultants, DGH-based consultants and those with a tertiary role (Chi-square test:χ42
 MathType@MTEF@5@5@+=feaafiart1ev1aaatCvAUfKttLearuWrP9MDH5MBPbIqV92AaeXatLxBI9gBaebbnrfifHhDYfgasaacH8akY=wiFfYdH8Gipec8Eeeu0xXdbba9frFj0=OqFfea0dXdd9vqai=hGuQ8kuc9pgc9s8qqaq=dirpe0xb9q8qiLsFr0=vr0=vr0dc8meaabaqaciaacaGaaeqabaqabeGadaaakeaacqaHhpWydaqhaaWcbaGaeGinaqdabaGaeGOmaidaaaaa@3078@ = 17.29, p = 0.002).

**Figure 3:**
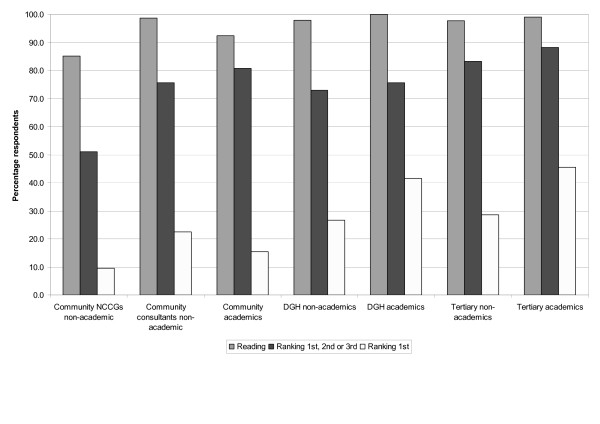
**Journals read by paediatricians and their importance to clinical practice**. The percentage of respondent paediatricians reading peer-reviewed journals and ranking them as important to inform their clinical practice.

Focusing specifically on peer-reviewed journals as an information source, Figure 3 shows the percentage reading them, the percentage ranking them first, second or third, and the percentage ranking them first for each of seven groups of paediatricians based on position held, academic responsibility and predominant role. Although journals are widely read by all groups the percentages of paediatricians within the groups who consider them first in importance show considerable variation. For community-based paediatricians at NCCG level the proportion is 10% but for academic, tertiary paediatricians, journals were considered first in importance by 46%.

### Journals read and considered important to clinical practice

For the number of journals read, the results reflect the greater importance of journals to academics than non-academics. 21% of respondents overall read three journals or less and 16% read 10 journals or more. Comparisons found significant differences at the 95% confidence level for the number of journals read by: consultants (mode 10+, median 6) versus NCCGs (mode 3, median 4, Chi-square test:χ92
 MathType@MTEF@5@5@+=feaafiart1ev1aaatCvAUfKttLearuWrP9MDH5MBPbIqV92AaeXatLxBI9gBaebbnrfifHhDYfgasaacH8akY=wiFfYdH8Gipec8Eeeu0xXdbba9frFj0=OqFfea0dXdd9vqai=hGuQ8kuc9pgc9s8qqaq=dirpe0xb9q8qiLsFr0=vr0=vr0dc8meaabaqaciaacaGaaeqabaqabeGadaaakeaacqaHhpWydaqhaaWcbaGaeGyoaKdabaGaeGOmaidaaaaa@3082@ = 97.85, p = < 0.001); academics (mode 10+, median 8) versus non-academics (mode 6, median 6, Chi-square test:χ92
 MathType@MTEF@5@5@+=feaafiart1ev1aaatCvAUfKttLearuWrP9MDH5MBPbIqV92AaeXatLxBI9gBaebbnrfifHhDYfgasaacH8akY=wiFfYdH8Gipec8Eeeu0xXdbba9frFj0=OqFfea0dXdd9vqai=hGuQ8kuc9pgc9s8qqaq=dirpe0xb9q8qiLsFr0=vr0=vr0dc8meaabaqaciaacaGaaeqabaqabeGadaaakeaacqaHhpWydaqhaaWcbaGaeGyoaKdabaGaeGOmaidaaaaa@3082@ = 70.43, p = < 0.001); and hospital-based consultants (mode 10+, median 7) versus community-based consultants (mode 3, median 5, Chi-square test:χ92
 MathType@MTEF@5@5@+=feaafiart1ev1aaatCvAUfKttLearuWrP9MDH5MBPbIqV92AaeXatLxBI9gBaebbnrfifHhDYfgasaacH8akY=wiFfYdH8Gipec8Eeeu0xXdbba9frFj0=OqFfea0dXdd9vqai=hGuQ8kuc9pgc9s8qqaq=dirpe0xb9q8qiLsFr0=vr0=vr0dc8meaabaqaciaacaGaaeqabaqabeGadaaakeaacqaHhpWydaqhaaWcbaGaeGyoaKdabaGaeGOmaidaaaaa@3082@ = 56.55, p = < 0.001).

Table 2 shows individual journals read by at least 20% of respondents in listed categories focusing on whether or not the respondents have academic responsibilities and their predominant role. *JAMA *is also included to allow comparisons across the seven journals included in the study by Birken et al. Whilst some journals e.g. *Archives of Disease in Childhood *and *BMJ*, are important across all groups, others are much more important to some than to others. For example, *Lancet *is read by 71% academics but only 39% non-academics and *Developmental Medicine and Child Neurology *is read by 69% community based paediatricians, excluding NCCGs, but only 16% of those with a tertiary role.

## Discussion

### Preferred information sources

In terms of the three measures used the overall picture across all respondent paediatricians indicated no one dominant source of information, but instead three were important. Furthermore, across all groups of paediatricians and for all situations studied, the picture was complex with no one source of information being the most important. Such findings highlight the need for careful analysis in terms of how to improve the flow of 'best evidence' to paediatricians.

The position of electronic databases perhaps highlights their potential for becoming increasingly important. They are down in sixth place for being consulted but, by both ranking measures used, they are placed fourth for importance. This might suggest that as their availability is increased, especially for paediatricians in the community, they will become an increasingly important source of information.

Riordan et al's study of information sources used by paediatricians-on-call in hospital units, mostly in training, found that guidelines and textbooks were most widely used and a few used the internet or journals. In our survey of the general use of information sources by consultants and NCCGs, journals were identified as, overall, the source ranked first, second or third most important by the highest number of paediatricians. In terms of being ranked first, however, 10% of non-academic community NCCGs ranked journals first, compared with 46% of tertiary academics doing so. Community NCCGs are also the only group for which medical education courses are ranked first by more paediatricians than are peer-reviewed journals (Figure 2). Clearly different dissemination strategies are likely to be most appropriate for the different groups and for different situations, but we have found that journals are confirmed as still being of considerable importance.

### Individual peer-reviewed journals

There was a significant difference between the numbers of individual journals read by hospital-based paediatricians and by those based in the community. This finding reflects the preference for peer-reviewed journals as a general information source and may reflect the greater availability of journals to clinicians in hospitals. Two membership journals, *Archives of Disease in Childhood *and *BMJ*, were the most widely read by all three groups (community-based, DGH-based and tertiary) and only one other – *Pediatrics *– is read by more than 20% of all three groups. It therefore appears that there are a small number of key journals for dissemination.

Birken et al [18] also suggested a large proportion (~ 40%–60%) of the best evidence for paediatric clinical practice was found in a small number of journals. They listed seven journals that they found to be in the top ten most cited by all three different sources of best evidence for paediatric clinical practice and Riordan et al found that all seven of these 'best-evidence' journals were available at 80% or more of the UK paediatrics and neonatal hospital units studied [7]. Overall, six out of these seven journals were the most widely read journals in our study (See Table 2) though in a different order to that suggested by Birken et al. A detailed analysis of the readership of the top six journals by different groups of paediatricians, reveals that all six journals are read by at least 40% of those who are DGH-based or tertiary, with or without academic commitments, but only two, *Archives Of Disease In Childhood *and *BMJ*, are read by more than 27% of any category of paediatricians based in the community. It appears then, that the variation in readership patterns for these seven journals containing 'best evidence' [18] is much greater than the variation in availability found in hospital units [7] and indeed the availability in the community is uncertain.

There was an even split (5:5) for the journals most widely read between those that are based in the UK and those that are based in the USA, with the top three based in the UK. This suggests a possible nationality bias but the issue of membership journals is a confounding factor.

Comparison of the findings from this survey of UK paediatricians with the survey of UK psychiatrists reveals many similarities [22]. These include: the importance of a small number of journals; the dominance of the main membership journal from the respective royal colleges; and the apparent prominence of UK-based journals.

This study, with a response rate of 43% and a mailing list restricted by the Medical Directories privacy policy, may not reflect the opinions of all paediatricians and the picture may be changing over time. Nevertheless, the survey was large and the response rate is comparable to rates previously obtained from similar studies of UK psychiatrists (47%) [22] and USA surgeons (38%) [6]. The findings add insight to the roles played by different information sources and journals, and could inform the debate on whether the assessment of clinical research should include some evaluation of the impact, or potential impact, made on clinicians. Further research investigating the information sources considered important by other professionals specialising in paediatrics and child health would widen the picture, thus providing information for a more comprehensive analysis.

## Conclusion

No one information source is dominant, therefore a variety of approaches to Continuing Professional Development should be used. Furthermore, given the variations different dissemination strategies for research findings are likely to be most appropriate for different groups of paediatricians. Overall, journals are an important information source for paediatricians and a small number of key journals can be identified, but the readership of specific ones varies within the specialty. By identifying the journals most read by clinicians to inform their clinical practice the findings could provide valuable additional input into the evaluation of clinical research outputs.

## Competing interests

The author(s) declare that they have no competing interests.

## Authors' contributions

MB and SH prepared grant submissions for the project, TJ, MB and SH were involved in the planning, preparation and approval of the original questionnaire. TJ conducted the questionnaire survey and data collection. TJ carried out the data analysis with additional intellectual input from MB and SH. All authors read and approved the final manuscript.

## Pre-publication history

The pre-publication history for this paper can be accessed here:



## Supplementary Material

Additional File 1 A study of which peer reviewed journals and other information sources are read or consulted on a regular basis and perceived as important by paediatricians to inform their clinical practice. The questionnaire distributed to UK paediatricians as part of the survey. Click here for file

## Figures and Tables

**Table 1:** The characteristics of respondent paediatricians in the questionnaire survey.

Position and academic responsibility	All (n = 993)	Community-based (n = 294)	DGH-based (n = 412)	Tertiary (n = 279)
Consultants	84%	60%	96%	96%
Non-consultant career grades	12%	34%	4%	1%
Other positions	4%	6%	1%	4%
				
With academic responsibility	18%	9%	10%	36%

**Table 2:** The journals most widely read by different groups of respondent paediatricians

	**All paediatricians (n = 993)**			**Tertiary (n = 279)**	**DGH-based (n = 412)**	**Community-based (n = 294)**	
	
**Journals read ^a^**	All	academic (n = 175)	non-academic (n = 807)	All	All	All, without NCCGs (n = 193)	^b^NCCGs (n = 101)
*Archives of Disease in Childhood (UK)*	96	97	96	95	99	97	92
*BMJ (UK)*	84	80	85	81	85	88	87
*Lancet (UK)*	45	71	39	62	50	22	13
*Pediatrics (USA)*	44	51	42	47	56	25	14
*Journal of Pediatrics (USA)*	43	55	40	53	54	24	7
*New England Journal of Medicine (USA)*	37	61	31	56	43	10	6
*Developmental Medicine & Child Neurology (UK)*	32	22	34	16	26	69	33
*Current Opinion in Pediatrics (USA)*	26	23	26	16	41	19	8
*Pediatric Clinics of North America (USA)*	21	17	21	13	33	13	7
Child Care, Health & Development (UK)	9	5	10	1	2	28	24

*JAMA *^c^	4	7	3	4	4	2	2

The table contains details of all journals read by at least 20% of paediatricians in one or more of the listed categories to inform their clinical practice.

^a^Journal names in italics indicate those included in the list on the original questionnaire;

^b^NCCGs, non-consultant career grades. NCCGs have been included separately for the Community-based paediatricians as they formed a large minority of the group (34%) whereas the numbers in the two hospital based groups were too small for separate analysis and have been included in the figures for all tertiary and all DGH-based paediatricians.

^C ^*JAMA *is included to provide a complete picture for the seven journals viewed as containing the 'best evidence' for paediatricians.

## References

[B1] Alper B, Hand J, Elliott S, Kinkade S, Hauan M, Onion D, Sklar B (2004). How much effort is needed to keep up with the literature relevant for primary care?. J Med Libr Assoc.

[B2] De Maeseneer J, van Driel M, Green L, van Weel C (2003). Research into practice II: The need for research in primary care. Lancet.

[B3] National Institute of Clinical Studies (2003). Information finding & assessment methods that different groups of clinicians find most useful.

[B4] Cullen R (1997). The medical specialist: information gateway or gatekeeper for the family practitioner. Bull Med Libr Assoc.

[B5] Dawes M, Sampson U (2003). Knowledge management in clinical practice: a systematic review of information seeking behavior in physicians. Int J Med Inform.

[B6] Schein M, Paladugu R, Sutija V, Wise L (2000). What American surgeons read: a survey of a thousand Fellows of the American College of Surgeons. Curr Surg.

[B7] Riordan F, Boyle E, Phillips B (2004). Best paediatric evidence; is it accessible and used on-call?. Arch Dis Child.

[B8] Hanney S, Mugford M, Grant J, Buxton M (2005). Assessing the benefits of health research: Lessons from research into the use of antenatal corticosteroids for the prevention of neonatal respiratory distress syndrome. Soc Sci Med.

[B9] Coomarasamy A, Gee H, Publicover M, Khan KS (2001). Medical journals and effective dissemination of health research. Hlth Information Lib.

[B10] Abbasi K (2004). Let's dump impact factors. BMJ.

[B11] Amin M, Mabe M (2000). Impact factors: use and abuse. Perspectives in Publishing.

[B12] Marcovitch H (2005). Submission to multiple journals to reduce publication times. BMJ.

[B13] Seglen PO (1997). Why the impact factor of journals should not be used for evaluating research. BMJ.

[B14] Williams G (1998). Misleading, unscientific, and unjust: the United Kingdom's research assessment exercise. BMJ.

[B15] Zetterstrom R (2002). Bibliometric data: a disaster for many non-American biomedical journals. Acta Paediatr.

[B16] Hanney S, Grant J, Wooding S, Buxton M (2004). Proposed methods for reviewing the outcomes of health research: the impact of funding by the UK's 'Arthritis Research Campaign'. Health Res Policy Syst.

[B17] Hughes E, Madden D, Linton J RAE 2008 Consultation on assessment panels' draft criteria and working methods. http://www.rae.ac.uk/pubs/2005/04/.

[B18] Birken CS, Parkin PC (1999). In which journals will pediatricians find the best evidence for clinical practice?. Pediatrics.

[B19] Dawson G, Lucocq B, Cottrell R, Lewison G (1998). Mapping the Landscape: National Biomedical Research Outputs 1988–95.

[B20] Thomson Scientific Philadelphia ISI Journal Citation Reports: JCR Glossary. http://scientific.thomson.com/products/jcr/.

[B21] Bowker R, Bowker R, ed (2003). Ulrich's international periodicals directory: including irregular serials and annuals.

[B22] Jones T, Hanney S, Buxton M, Burns T (2004). What British psychiatrists read. Questionnaire survey of journal usage among clinicians. Br J Psychiatry.

