# Supplementary material for: The information sources and journals consulted or read by UK paediatricians to inform their clinical practice and those which they consider important: a questionnaire survey
Source: BMC Pediatr. 2007 Jan 15;7:1. doi: 10.1186/1471-2431-7-1 (PMC1783849; doi:10.1186/1471-2431-7-1)
Supplement: Additional File 1 — A study of which peer reviewed journals and other information sources are read or consulted on a regular basis and perceived as important by paediatricians to inform their clinical practice. The questionnaire distributed to UK paediatricians as part of the survey. [file 1471-2431-7-1-S1.doc]

abcdefgh

A study of which peer reviewed journals and other information sources are read or consulted on a regular basis and perceived as important by paediatricians to inform their clinical practice.

*This questionnaire can be completed in about 5 minutes. Your answers will be entirely anonymous: there is no identification number on the questionnaire or the reply-paid envelope.*

**Please return the completed questionnaire to us in the envelope provided or to the address below.**

*Thank you for your time and co-operation.*

*Further details about the background to the project are supplied on the last page but if you have any questions about the survey please contact:*

Teri Jones or Dr Steve Hanney
Health Economics Research Group

Brunel University

Uxbridge, Middlesex. UB8 3PH

Telephone 01895 265445 or 265444
Email: [teresa.jones@brunel.ac.uk](mailto:teresa.jones@brunel.ac.uk), stephen.hanney@brunel.ac.uk

1. What position do you hold?

Consultant	☐ Non-Consultant Career Grade	☐Other	☐
*Please specify*………………………

2. How many sessions per week do you work?

Clinical	…...

Academic	……

3. Is your role predominantly:

Community based	☐District General Hospital

based	☐

Tertiary	☐

Other	☐*Please specify*………………………

4. In which sub-specialties do you work? (Please tick as many as necessary)

Clinical genetics	☐
Community child health	☐
Neonatal medicine	☐
Paediatric cardiology	☐
Paediatric endocrinology	☐
Paediatric gastroenterology	☐
Paediatric immunology &

infectious diseases	☐

Paediatric nephrology	☐
Paediatric neurology	☐
Paediatric oncology	☐
Paediatric respiratory

medicine	☐
Paediatric rheumatology	☐
Other	☐
*Please specify ........................................................*

5. **Please tick any information sources that you consult or attend *to inform your clinical practice.* If necessary add any that are not listed*.* From those you have ticked, please *rank* the top three (i.e. 1 for first, 2 for second or 3 for third) in terms of their importance in informing your clinical practice.**

| **Information Sources** | **Tick** | **Rank Top 3** |
| --- | --- | --- |
| Medical Colleagues |  |  |
| Professional meetings/conferences |  |  |
| Medical education courses |  |  |
| Journals: Peer-reviewed |  |  |
| Journals: Non peer-reviewed |  |  |
| Textbooks & Compendia: Traditional book form |  |  |
| Textbooks & Compendia: Electronic form |  |  |
| Grey literature (eg Documents from the Royal College, Medicines for Children, Handbooks eg from societies, individuals etc.) |  |  |
| Commercial/industrial literature |  |  |
| Newspapers and magazines |  |  |
| Electronic databases (eg Cochrane Updates) |  |  |
| Others – please name |  |  |
|  |  |  |
|  |  |  |
|  |  |  |

6. Please tick up to ten journals in total that you read or consult on a regular basis *to inform your clinical practice.* If necessary add any that are not listed*.* From those you have ticked, please *rank* the top three journals (i.e. 1 for first, 2 for second or 3 for third) in terms of their importance in informing your clinical practice.

| Journal | Tick up to 10 | **Rank Top 3** |
| --- | --- | --- |
| Acta Paediatrica |  |  |
| Annals of Internal Medicine |  |  |
| Annual Review of Medicine |  |  |
| Archives of Disease in Childhood |  |  |
| Archives of Pediatrics & Adolescent Medicine |  |  |
| Birth: Issues in Perinatal Care |  |  |
| British Dental Journal |  |  |
| British Journal of Cancer |  |  |
| British Journal of Haematology |  |  |
| British Journal of Obstetrics and Gynaecology |  |  |
| BMJ |  |  |
| Current Opinion in Pediatrics |  |  |
| Developmental Medicine and Child Neurology |  |  |
| European Journal of Pediatrics |  |  |
| European Respiratory Journal |  |  |
| International Journal of Pediatric Otorhinolaryngology |  |  |
| Journal of Adolescent Health |  |  |
| Journal of Child & Adolescent Psychopharmacology |  |  |
| Journal of Developmental & Behavioral Pediatrics |  |  |
| Journal of Pediatric Gastroenterology and Nutrition |  |  |
| Journal of Pediatric Surgery |  |  |
| Journal of Pediatrics |  |  |
| Journal of the American Academy of Child & Adolescent Psychiatry |  |  |
| Journal of the American Medical Association |  |  |
| Journal of the Royal Society of Medicine |  |  |
| Lancet |  |  |
| Medical and Pediatric Oncology |  |  |
| Mental Retardation & Developmental Disabilities Research Reviews |  |  |
| New England Journal of Medicine |  |  |
| Paediatric and Perinatal Epidemiology |  |  |
| Pediatric Allergy and Immunology |  |  |
| Pediatric Clinics of North America |  |  |
| Pediatric Infectious Disease Journal |  |  |
| Pediatric Pulmonology |  |  |
| Pediatric Research |  |  |
| Pediatrics |  |  |
| Prenatal Diagnosis |  |  |
| Seminars in Perinatology |  |  |
| Ultrasound in Obstetrics & Gynecology |  |  |
|  |  |  |
|  |  |  |
|  |  |  |
|  |  |  |
|  |  |  |

Thank you for your time and co-operation.

**Background:** Clinical research generally does not command the same level of citation as basic research. The assessment of research quality often uses the journal impact factor, based on the average citation rate of the journal, but clinical research is less likely to appear in a journal with a high impact factor. Too often the result is the *apparent* poor performance of establishments or units involved in clinical research relative to those concentrating on research of a more basic nature. Our research is attempting to identify the information sources that are important to clinical practitioners and, where appropriate, compare the findings with journal impact factors.

**Derivation of the list of journals:** Two sources were drawn upon to produce the single list of journals presented in Question 6 in alphabetical order:

- *Journal Citation Reports 2002 (JCR) from ISI:* Ranked by journal impact factor, the top 20 journals from the field of paediatrics were combined with the top 5 from the general medical category.
- *The Research Outputs Database (ROD) that was originally constructed by The Wellcome Trust*: A subset of ROD (NHS ROD) was compiled containing details of publications from England that involve some element of NHS financial input. The top 20 journals were extracted containing the most publications on the NHS ROD related to paediatrics and neonatology over the period 1997-2001.
